# Outcomes of a multicenter registry on EUS-guided gallbladder drainage as a rescue technique for malignant distal biliary obstruction after failed endoscopic retrograde cholangiopancreatography

**DOI:** 10.1097/eus.0000000000000116

**Published:** 2025-05-05

**Authors:** Belén Martínez-Moreno, Gonzalo López-Roldán, Julia Escuer, Joan B. Gornals, Carme Loras, Ana Gordo, Juan Vila, Sergio Bazaga, Miguel Durá, Vicente Sanchiz, Natividad Zaragoza, Ferrán Gonzalez-Huix, Alejandro Repiso, José Ramón Aparicio

**Affiliations:** 1 Hospital General Universitario Dr. Balmis, Alicante, Spain; 2 Instituto de Investigación Sanitaria y Biomédica de Alicante (ISABIAL), Alicante, Spain; 3 Hospital Universitari de Bellvitge, Universidad de Barcelona, L'Hospitalet, Barcelona, Spain; 4 Bellvitge Biomedical Research Institute (IDIBELL), Barcelona, Spain; 5 Hospital Universitari Mútua Terrassa, Terrassa, Spain; 6 Centro de Investigación Biomédica en Red de Enfermedades Hepáticas y Digestivas (CIBERehd), Madrid, Spain; 7 Complejo Hospitalario de Navarra, Pamplona, Spain; 8 Hospital General de Granollers, Granollers, Spain; 9 Hospital Universitario de Cruces, Barakaldo, Spain; 10 Hospital Clínico Universitario de Valencia, Valencia, Spain; 11 Hospital Universitario Arnau de Vilanova, Lleida, Spain; 12 Clínica Girona, Girona, Spain; 13 Hospital Universitario de Toledo, Toledo, Spain.

**Keywords:** EUS, Therapeutic EUS, Gallbladder, MDBO, LAMS, ERCP, EUS-guided biliary drainage

## Abstract

**Background and Objectives:**

Endoscopic retrograde cholangiopancreatography (ERCP) is the primary intervention for malignant distal biliary obstruction (MDBO). However, ERCP may fail for various reasons, requiring alternative interventions such as EUS-guided biliary drainage. Among EUS-guided biliary drainage (EUS-BD) methods, EUS-guided gallbladder drainage (EUS-GBD) is emerging as a viable option for patients who have failed ERCP and EUS-BD. The aim of this study is to evaluate the efficacy and safety of EUS-GBD as salvage therapy for MDBO and its potential role in allowing the initiation of chemotherapy.

**Methods:**

This is a retrospective multicenter study of consecutive patients with MDBO with failed ERCP and/or EUS-BD that subsequently underwent EUS-GBD with lumen-apposing metal stent.

**Results:**

Ninety-six patients from 9 centers in Spain were included. Technical success was achieved in 99% of patients, while clinical success, defined as bilirubin reduction <50% within 14 days after the procedure, was achieved in 78.1% of patients. Bilirubin levels were normalized in 65.6% of patients. The median time to normalization of bilirubin levels was 15 (7–27) days. Related to continuation of oncological treatment, 44/77 (57.1%) eligible patients were able to start chemotherapy after the procedure, and 12/17 (70.6%) eligible patients underwent surgery in the end. Adverse events were observed in 26.3% of cases, with 3 patients requiring surgery and 3 deaths related to EUS-GBD.

**Conclusions:**

EUS-GBD represents a potential alternative to MDBO in cases where ERCP has failed, with an appropriate profile of patients starting chemotherapy. However, in light of the considerable number of adverse events and the moderate efficacy, it may be advisable to consider this approach as a second-line option.

## INTRODUCTION

Endoscopic retrograde cholangiopancreatography (ERCP) is the standard of care for patients with malignant distal biliary obstruction (MDBO).^[1]^ There are several potential reasons for the failure of ERCP, including altered anatomy, inability to reach the papilla, and inability to achieve deep cannulation. This can occur in 16% of cases in malignant biliary obstruction.^[2]^ Nowadays, EUS-guided biliary drainage (EUS-BD) is an accepted alternative when ERCP fails and dedicated experts are available.^[3]^ EUS-BD can be performed by EUS-guided choledochoduodenostomy or EUS-guided hepaticogastrostomy. Occasionally, the bile duct is not sufficiently dilated to safely perform a biliary drainage. Common bile ducts <13 to 14 mm in diameter are difficult to drain with lumen-apposing metal stent (LAMS), and nondilated intrahepatic bile ducts are not easily punctured with an EUS-guided fine-needle aspiration needle. Therefore, up to 7% of patients with MDBO may fail to undergo EUS-BD.^[4]^ In these patients, if the cystic duct is patent, the gallbladder is usually distended, making it an easy target. In those cases, EUS-guided gallbladder drainage (EUS-GBD) appears to be a possible salvage therapy.

The European Society of Gastrointestinal Endoscopy guidelines suggest that EUS-GBD may be performed in this situation as a rescue procedure for patients who have failed ERCP and EUS-BD.^[3]^ However, data on this procedure remain scarce. The aim of this study was to assess the efficacy and safety of EUS-GBD as rescue therapy for MDBO, with a particular focus on its efficacy in initiating chemotherapy, in a large series of patients from multiple centers in Spain.

## MATERIALS AND METHODS

This is a multicenter retrospective study from 9 tertiary centers in Spain. The study included patients with MDBO who had failed attempts at ERCP and EUS-BD, followed by EUS-GBD, between May 2016 and May 2024. The inclusion criteria were as follows: age ≥18 years, patent cystic duct on EUS above the stricture, accessible GB for the drainage from the stomach or duodenum, and previous failed ERCP. Patients were excluded if they had malignant cystic duct involvement as documented by EUS, surgically altered upper gastrointestinal anatomy, international normalized ratio >1.5, or a platelet count <50,000/μL.

Technical success was defined as the successful deployment of the stent between the gallbladder lumen and the stomach or duodenum. Clinical success was defined as a reduction in bilirubin levels of at least 50% of the pretreatment level within 2 weeks after the procedure. The normalization of bilirubin levels was considered when the bilirubin levels dropped below 3 mg/dL.

Adverse events (AEs) were classified in accordance with the AGREE system.^[5]^ Intraprocedural events were defined as those that occurred during the performance of the endoscopic drainage procedure. The term “early adverse events” was used to describe those occurring within 30 days of stent implantation, whereas the term “late adverse events” was used to describe those occurring after this period. Stent dysfunction was defined as the requirement any time for endoscopic, surgical, or percutaneous procedures to relieve biliary symptoms in patients achieving clinical success. These included the following: migration, perforation, stent occlusion, gastric outlet obstruction related to the stent, buried stent, and biliary infection.

**Figure 1:**
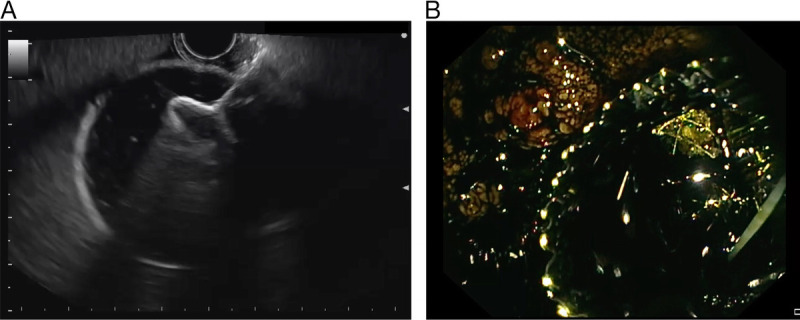
EUS-guided gallbladder drainage procedure. A, Delivery of the distal flange of the LAMS. B, Outflow of retained bile through the LAMS. LAMS: lumen-apposing metal stent.

EUS-GBD was performed using a linear-array echoendoscope with carbon dioxide insufflation under deep sedation with propofol and the direct supervision of the endoscopy team or under general anesthesia. The use of color flow Doppler imaging allowed for the avoidance of intervening blood vessels. Following the exclusion of cystic duct obstruction, an electrocautery-enhanced LAMS was used (Figure 1). Once the delivery catheter was positioned within the gallbladder and the initial flange was deployed under EUS guidance, the second flange was released using the intrachannel technique. The decision regarding the dimensions of the LAMS, as well as the size and placement of the double-pigtail plastic stent within the LAMS, was at the discretion of the endoscopist. Continuous variables are reported as mean (SD) or as median and interquartile range (IQR), and categorical variables are summarized as frequencies and percentages. Comparisons of variables were made using the *t* test or *χ^2^* test as appropriate. A *P* < 0.05 was considered statistically significant. All statistical analyses were performed using SPSS version 28.0 for Macintosh (SPSS Inc, Chicago, Illinois).

## RESULTS

A total of 96 patients were enrolled over the study period from 9 centers in Spain. Of these, 53 were women (55.2%), with a mean (SD) age of 72 (13.4) years. Demographic and clinical characteristics are shown in Table 1. In all cases, EUS-GBD was performed during the same session as the unsuccessful ERCP. The most common indication for the technique was failed EUS-BD and/or rendezvous (43 cases, 45%), followed by operator preference over other EUS-BDs (38 cases, 40%), lack of biliary dilatation to perform EUS-BD (10 cases, 10%), and associated cholecystitis (5 cases, 5%). In all patients, a Hot Axios (Boston Scientific) LAMS was utilized for the performance of the EUS-GBD. The most commonly used stent size was 10 mm × 10 mm in 49 (51%) patients and 15 mm × 10 mm in 31 (32%) patients. The remaining sizes used were 8 mm × 8 mm in 14 (15%) patients and 6 mm × 8 mm in 2 (2%) patients. In 57 cases (59%), a coaxial pigtail was inserted. LAMSs were placed in 41 cases (43%) transduodenally and in 55 cases (57%) transgastrically. The median follow-up period was 72 days (IQR, 25–154 days).

**Table 1:** Characteristics of patients.

Characteristics	*n* = 96 (%)
Age (yr)	72 ± 12
Female gender	53 (55)
ASA II/III/IV	19/73/4
Charlson index	8 ± 3
Presence of ascites	29 (30)
Reason for EUS-GBD	
Failed EUS-BD or RV	43 (45)
Operator preference	38 (40)
No biliary dilatation	10 (10)
Associated cholecystitis	5 (5)
Size of LAMS (mm^2^)	
15 × 10	31 (32)
10 × 10	49 (51)
8 × 8	14 (15)
6 × 8	2 (2)
Coaxial pigtail	57 (59)

ASA: American Society of Anesthesiologists; EUS-BD: EUS-guided biliary drainage; EUS-GBD: EUS-guided gallbladder drainage; LAMS: Lumen-apposing metal stent; RV: Rendezvous.

Technical success was achieved in 95 of the 96 patients (98.9%; 95% confidence interval [CI], 96.8%–100%). The only patient who experienced a technical failure suffered an intraprocedural dislodgement of the LAMS, which resulted in the need for urgent surgical intervention.

Clinical success was achieved in 75 of the 95 patients (78.9%; 95% CI, 70.7%–87.1%). No correlation was observed between clinical success and the size of the LAMS, the utilization of a coaxial pigtail, or the access route.

Normalization of bilirubin levels was observed in 63 patients (66.3%; 95% CI, 56.8%–75.8%). The median time to normalization of bilirubin levels was 15 days (IQR, 7–27 days).

Of the 77 patients who were eligible for adjuvant therapy, 44 (57.1%; 95% CI, 46%–68.1%) were finally able to start chemotherapy. When clinical success was achieved, 40/59 (67.8%) patients received chemotherapy versus 4/17 (23.5%) patients after unsuccessful drainage, *P* = 001. Similarly, 38/53 (71.7%) patients with normalized bilirubin levels received chemotherapy versus 6/23 (26.1%) patients who did not normalize bilirubin levels, *P* < 0.001.

Of the 17 patients who were eligible for surgical intervention, 12 underwent surgery (70.6%), with no evidence of stent-related surgical difficulty in any case.

**Figure 2:**
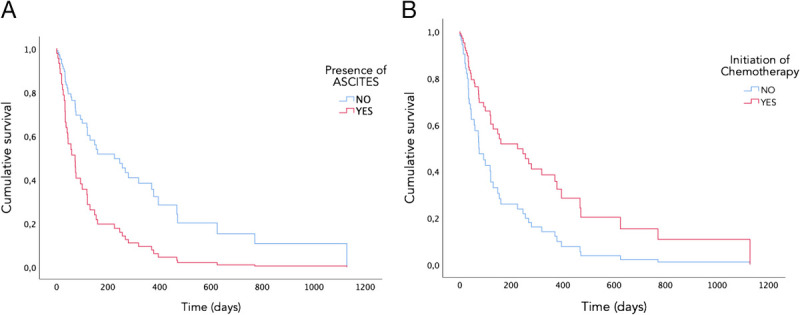
Comparison of overall survival between groups of patients according to (A) presence of ascites and (B) presence of chemotherapy.

The Kaplan-Meier survival analysis revealed a statistically significant correlation between mortality rates and the following factors: normalization of BT (*P* = 0.007), the presence of ascites (*P* < 0.001), American Society of Anesthesiologists classification (*P* = 0.043), and the initiation of chemotherapy (*P* < 0.001). The results of the Cox regression multivariate analysis indicated that the presence of ascites (hazard ratio [HR], 2.5; 95% CI, 1.2–4.9; *P* = 0.009) and the possibility of initiating chemotherapy (HR 0.5; 95% CI, 0.2–0.9; *P* = 0.046) were the only factors that remained statistically significant (Figure 2).

### Adverse events

Twenty-five patients experienced AEs (26.3%), with the most frequent being infection in 8 patients, stent migration in 5 patients, and stent occlusion in 4 patients (Table 2). The severity of the adverse effects was classified as grade I–II in 5 patients (5.3%), grade IIIa in 16 patients (16.8%), grade IIIb in 2 patients (2.1%), and grade V in 2 patients (2.1%). The majority of AEs occurred early (68%) with a median (IQR) of 16 (7–78) days. Table 1 of the Supplementary material provides a comprehensive overview of all adverse effects, http://links.lww.com/ENUS/A372.

**Table 2:** Outcomes of patients with technical success.

Variables	*n* = 95 (%)
Clinical success	75 (78.9)
Bilirubin normalization	63 (66.3)
Started CTx	44/77 (57.1)
Operated	12/17 (70.6)
Adverse events	25 (26)
Infection	8 (8.3)
Stent migration	5 (5.2)
Stent occlusion	4 (4.2)
Bleeding	3 (3.1)
Buried stent	2 (2.1)
GOO	1 (1.0)

CTx: Chemotherapy; GOO: Gastric outlet obstruction.

Of the AEs observed, those related to stent dysfunction that motivated reintervention occurred in 17 patients, representing an overall reintervention rate due to stent dysfunction of 18%. Dysfunction of the stent occurred in 5 cases due to infection with 3 documented cystic duct occlusions and 2 cases of bacteremia. Additionally, further stent dysfunctions occurred in 4 cases due to stent migration, 2 buried stents, 1 case due to gastric outlet obstruction, and in 1 patient due to perforation.

Twelve of the 17 patients (70.6%) were managed endoscopically. This included the performance of a hepaticogastrostomy in all 3 cases of cystic obstruction. Another patient was managed with percutaneous drainage, whereas 1 patient with perforation was managed conservatively due to their poor basal condition. The remaining 3 patients were managed surgically.

During the follow-up period, 5 patients (5.3%) experienced partial or complete migration of the LAMS at the 5th, 8th, 11th, 91st, and 218th days after the procedure. Of these cases, the 3 earliest migrations (within the first 14 days) required surgical intervention due to perforation or choleperitoneum. One of these cases resulted in a fatal outcome. On the contrary, late migrations could be managed conservatively in 1 case of internal migration, and with placement of another EUS-GBD LAMS in the other patient. Our analysis revealed no correlation between the stent size or location and the occurrence of stent migration.

Of the AEs related to the stent procedure, the only patient who experienced an intraprocedural event died as a result of postoperative bronchoaspiration. In the follow-up, 3 patients presented with a possible or probable stent-related death: 1 case of infection was managed conservatively due to the poor patient’s condition, 1 perforation and 1 migration occurred 24 hours and 5 days after the procedure, respectively. Therefore, the overall EUS-GBD–related death rate was 4.2%.

## DISCUSSION

At present, EUS-GBD represents a well-established option for the management of patients with nonsurgical acute cholecystitis. Good long-term results have been reported when the stent is left in place indefinitely.^[6,7]^ These favorable outcomes have raised the consideration of performing EUS-GBD in other indications. Currently, the European Society of Gastrointestinal Endoscopy guidelines suggest that EUS-GBD may be considered as a salvage procedure in patients with inoperable MDBO when ERCP and EUS-BD have failed and cystic duct patency is confirmed.^[3]^ The fact that the drainage of a distended gallbladder is technically much less demanding than the drainage of the common bile duct or performing a hepaticogastrostomy is the reason for the great interest of the endoscopic community in this technique as a possible drainage technique in cases of MDBO.

The use of the gallbladder as a route to relieve biliary obstruction in cases of MDBO is not a new concept. Surgical cholecystojejunostomy has been used to relieve neoplastic obstructive jaundice since the 1960s. Nevertheless, in surgical series following cholecystojejunostomy, 11% of patients failed to resolve the jaundice, and 15% experienced a recurrence. Overall, 24% of patients had treatment failure.^[8]^ The essential requirement is that the cystic duct must be patent. However, in the study by Tarnasky et al.,^[8]^ only half of the patients with neoplastic obstructive jaundice had a cystic duct that was permeable on ERCP. Furthermore, in 56% of cases where the cystic duct was permeable, the neoplastic obstruction was less than 1 cm away, indicating that it would likely be affected during follow-up. These modest results differ from those of the reported case series of EUS-GBD as a rescue drainage. The results of the most relevant studies published on this subject are shown in Table 3.^[9–13]^

**Table 3:** Summary of evidence including published studies of EUS-GBD with EC-LAMS.

Authors, year	*n*	Type study	Type stent	Technical success	Clinical success	Start CTx	AE rate	Reintervention rate*
Paleti et al.,^[7]^ 2019	7	Retrospective	EC-LAMS	100%	100%	—	0%	—
Chang et al.,^[8]^ 2019	9	Retrospective	EC-LAMS	100%	77.8%	—	0%	0%
Binda et al.,^[9]^ 2023	48	Multicenter retrospective	EC-LAMS	100%	81.3%	28%	10.4%	2/47 (4.3%)
Mangiavillano et al.,^[10]^ 2024	37	Prospective	EC-LAMS	100%	100%	No CTx candidates	10.8%	3/37 (8.1%)
Debourdeau et al.,^[11]^ 2025	41	Multicenter retrospective	EC-LAMS	100%	87.8%	47.4%	7.3%	5.9%
Martínez-Moreno et al.,^[12]^ 2024	96	Multicenter retrospective	EC-LAMS	98.9%	78.9%	66.3%	26%	18%

*Reintervention rate due to stent malfunction.

AE: Adverse event; CTx: Chemotherapy; EC-LAMS: Electrocautery lumen-apposing metal stent; EUS-GBD: EUS-guided gallbladder drainage; EUS-GBD: EUS-guided gallbladder drainage.

In these studies, technical success was achieved in all cases, with clinical success rates ranging from 77.8% to 100% and AEs ranging from 0% to 10.8%. A recent meta-analysis also showed good results with the use of LAMS in this indication, with 85% clinical success, 13% AEs, and a 9% reintervention rate.^[14]^

To date, our study represents the longest series of publications on this topic, with a total of 96 patients included. However, in contrast to the results of previous studies, our study shows a slightly lower clinical success rate of 78.9%, with a significant increase in the incidence of AEs of 26%, including 3 patients who required urgent surgical intervention after an AE. The high rate of AE observed in this study differs from the AE reported for EUS-GBD in acute cholecystitis, which has been documented at 18% after the first year (^[7]^ in similar patient cohorts. In a large meta-analysis involving 546 patients, the AE was even lower at 12.4%.^[15]^ Furthermore, there have been no reports of early migrations under this indication.

We postulate that the reason for this discrepancy is that, in contrast to acute cholecystitis, the absence of inflammation may not promote the formation of adhesion between the gallbladder and the stomach or duodenum. Furthermore, in MBDO cases, it is not uncommon to observe a markedly distended gallbladder, which, following drainage, rapidly collapses and empties. This may result in a change to the distance and an increase in tension between the gallbladder wall and the duodenum/stomach wall, which could potentially lead to early dislocation of a flap of the LAMS.

In our series of patients, 17% required reintervention due to stent dysfunction. The primary reason for a reintervention was infection. Three cases of cholangitis developed following cystic occlusion, and 2 instances of bacteremia occurred after stent migration and stent occlusion. Whereas 70% of patients who underwent reintervention could be managed endoscopically, 3 patients required surgery, and 1 patient was managed conservatively due to poor condition.

Therefore, despite data suggesting this technique as a potential first-line treatment after failed ERCP or even as an alternative to ERCP, our analysis calls for caution in the indication of this technique due to the nonnegligible possibility of significant AEs.

On the other hand, there is a lack of data on the efficacy and outcomes of EUS-GDB, in relation to the potential for initiating chemotherapy treatment following drainage, which should be one of the main goals of biliary drainage in oncological patients.

In order to address the real oncological benefit of EUS-GBD, our study represents the first investigation of EUS-GBD as a rescue drainage therapy in MDBO that assesses the normalization of bilirubin levels, the time required for this normalization, and the rate of patients who ultimately started chemotherapy. In our series, 66.3% of patients achieved normal bilirubin levels with a median time to normalization of 15 (7–27) days, and 57.1% of candidates were finally able to start chemotherapy.

Our series demonstrates that chemotherapy initiation is a significant protective factor for survival, with an HR of 0.5. Furthermore, clinical success and normalized bilirubin were found to be significantly associated with the possibility of chemotherapy initiation. Previous studies after percutaneous biliary drainages have reported similar results, with a probability of 72.7% of patients receiving chemotherapy after stenting versus only 25% of patients with unsuccessful drainages.^[16]^

Our results indicate that the only factor that can be influenced and has an impact on the reduction of mortality is the ability to commence chemotherapy. This can only occur when bilirubin levels are at a very low or normalized level. However, the standard definition used in most studies for clinical success is a decrease in bilirubin levels to below 50% of baseline at 14 days, which may not be sufficient to commence further treatments. We believe that future studies evaluating different endoscopic techniques for biliary drainage should focus on clinically meaningful oncological outcomes, such as bilirubin normalization rates, time to normalization, and the rate of patients who are candidates for chemotherapy who finally succeed in initiating chemotherapy or undergo surgery. This will ensure an adequate assessment of the clinical efficacy and true disease impact of the techniques being used in these patients and allow for effective comparison between the various techniques.

It is also worth emphasising that 12 of the 17 suitable candidates (70%) ultimately underwent surgical intervention and that all procedures were completed without any complications. This reinforces the possibility of performing this technique in the context of resectable or potentially resectable stages of disease. However, further studies would be required to address this topic in more detail.

In conclusion, EUS-GBD appears to be a moderately effective drainage technique in MDBO cases, enabling patients to proceed with chemotherapy or surgery at an acceptable rate. However, the considerable number of significant adverse effects associated with the procedure makes it unlikely that it will become the primary drainage technique. EUS-GBD would be recommended only in rescue cases and when no other endoscopic technique is feasible.

## Acknowledgments

None.

## Source of Funding

No funding was received for this study.

## Ethical Approval

A registry of therapeutic procedures has been approved by the Ethics Committee of the University Hospital Dr. Balmis of Alicante (No. 2024-0552).

## Informed Consent

Informed consent was obtained from all patients.

## Conflicts of Interest

Belén Martínez-Moreno and José Ramón Aparicio are consultants to Boston Scientific consultant. The other authors declare that they have no financial conflict of interest with regard to the content of this report.

## Author Contributions

Belén Martínez-Moreno participated in research design, data analysis, and writing of the paper. José Ramón Aparicio participated in research design, data analysis, and critical revision of the paper. Gonzalo López-Roldán participated in research design and performance of the research. Joan B. Gornals, Sergio Bazaga, and Miguel Durá participated in performance of the research and critical revision of the paper. Julia Escuer, Carme Loras, Ana Gordo, Juan Vila, Vicente Sanchiz, Natividad Zaragoza, Ferrán Gonzalez-Huix, and Alejandro Repiso participated in performance of the research.

## Data Availability Statement

The datasets generated and analyzed during the current study are available from the corresponding author on reasonable request.

## References

[1] Dumonceau JM Tringali A Papanikolaou IS, . Endoscopic biliary stenting: indications, choice of stents, and results: European Society of Gastrointestinal Endoscopy (ESGE) clinical guideline—updated October 2017. *Endoscopy* 2018;50(9):910–930. doi:10.1055/a-0659-9864. 30086596

[2] Enochsson L Swahn F Arnelo U Nilsson M Löhr M Persson G. Nationwide, population-based data from 11,074 ERCP procedures from the Swedish Registry for Gallstone Surgery and ERCP. *Gastrointest Endosc* 2010;72(6):1175–1184.e11843. doi:10.1016/j.gie.2010.07.047. 20970787

[3] Van der Merwe SW van Wanrooij RLJ Bronswijk M, . Therapeutic endoscopic ultrasound: European Society of Gastrointestinal Endoscopy (ESGE) guideline. *Endoscopy* 2022;54(2):185–205. doi:10.1055/a-1717-1391. 34937098

[4] Issa D Irani S Law R, . Endoscopic ultrasound–guided gallbladder drainage as a rescue therapy for unresectable malignant biliary obstruction: a multicenter experience. *Endoscopy* 2021;53(8):827–831. doi:10.1055/a-1259-0349. 32898918

[5] Nass KJ Zwager LW van der Vlugt M, . Novel classification for adverse events in GI endoscopy: the AGREE classification. *Gastrointest Endosc* 2022;95(6):1078–1085.e8. doi:10.1016/j.gie.2021.11.038. 34890695

[6] Teh JL Rimbas M Larghi A Teoh AYB. Endoscopic ultrasound in the management of acute cholecystitis. *Best Pract Res Clin Gastroenterol* 2022;60-61:101806. doi:10.1016/j.bpg.2022.101806. 36577527

[7] Martinez-Moreno B López-Roldán G Martínez-Sempere J de-Madaria E Jover R Aparicio JR. Long-term results after EUS gallbladder drainage in high-surgical-risk patients with acute cholecystitis: a 3-year follow-up registry. *Endosc Int Open* 2023;11(11):E1063–E1068. doi:10.1055/a-2180-9817. 37954111 PMC10637859

[8] Tarnasky PR England RE Lail LM Pappas TN Cotton PB. Cystic duct patency in malignant obstructive jaundice. An ERCP-based study relevant to the role of laparoscopic cholecystojejunostomy. *Ann Surg* 1995;221(3):265–271. doi:10.1097/00000658-199503000-00008. 7536405 PMC1234568

[9] Paleti S Gulati R Sánchez-Luna SA Ling C Rustagi T. Su1191 EUS-guided gall bladder drainage using lumen apposing metal stent for malignant biliary obstruction. *Gastrointest Endosc* 2019;89(6):AB308. doi:10.1016/j.gie.2019.03.1212.

[10] Chang JI Dong E Kwok KK. Endoscopic ultrasound–guided transmural gallbladder drainage in malignant obstruction using a novel lumen-apposing stent: a case series (with video). *Endosc Int Open* 2019;7(5):E655–E661. doi:10.1055/a-0826-4309. 31058208 PMC6497498

[11] Binda C Anderloni A Fugazza A, . EUS-guided gallbladder drainage using a lumen-apposing metal stent as rescue treatment for malignant distal biliary obstruction: a large multicenter experience. *Gastrointest Endosc* 2023;98(5):765–773. doi:10.1016/j.gie.2023.06.054. 37392954

[12] Mangiavillano B Moon JH Facciorusso A, . Endoscopic ultrasound–guided gallbladder drainage as a first approach for jaundice palliation in unresectable malignant distal biliary obstruction: prospective study. *Dig Endosc* 2024;36(3):351–358. doi:10.1111/den.14606. 37253185

[13] Debourdeau A Daniel J Caillo L, . Effectiveness of endoscopic ultrasound (EUS)–guided choledochoduodenostomy vs. EUS-guided gallbladder drainage for jaundice in patients with malignant distal biliary obstruction after failed endoscopic retrograde cholangiopancreatography: retrospective, multicenter study (GALLBLADEUS study). *Dig Endosc* 2025;37(1):103–114. Published online 2024. doi:10.1111/den.14750. 38380564 PMC11718144

[14] Kamal F Khan MA Lee-Smith W, . Efficacy and safety of EUS-guided gallbladder drainage for rescue treatment of malignant biliary obstruction: a systematic review and meta-analysis. *Endosc Ultrasound* 2023;12(1):8–15. doi:10.4103/EUS-D-21-00206. 36861505 PMC10134926

[15] Mohan BP Khan SR Trakroo S, . Endoscopic ultrasound–guided gallbladder drainage, transpapillary drainage, or percutaneous drainage in high risk acute cholecystitis patients: a systematic review and comparative meta-analysis. *Endoscopy* 2020;52(2):96–106. doi:10.1055/a-1020-3932. 31645067

[16] Sellier F Bories E Sibertin-Blanc C, . Clinical outcome after biliary drainage for metastatic colorectal cancer: survival analysis and prognostic factors. *Dig Liver Dis* 2018;50(2):189–194. doi:10.1016/j.dld.2017.09.121. 29054396

